# Event and Comment

**Published:** 1920-09

**Authors:** 


### DENTAL REGISTER

THE MAGAZINE OF DENTISTRY

Vol. LXXIV SEPTEMBER, 1920 No. 9

### EVENT AND COMMENT

**Dental Prophylaxis and Hygiene** During the past decade there has been a great advancement in our knowledge of the health situation in its relations to the teeth. There has been much discussion of dental matters in respect to their usefulness and conservation, from development to artificial restoration, technically as well as biologically and pathologically.

More recently we seem to have focused our observations, and intensified our studies on the influences which the teeth exert as centers of or foci of ill health, of both the general and of the local diseases and their sympathetic sequelae.

It will be observed that the causes of most diseases of oral significance have in practically all cases been of slow growth, and their intensity have been complicated by local environments. It will also be observed that lack of proper oral sanitation and lack of intelligent use of the functions of mastication have an important influence in causing local dental diseases. It is a matter of common observation that in childhood the first teeth degenerate when their teeth fail to function because of disease, either in body or environment. The teeth must be used to their capacities, and the gingival environments must be normal. If not practicable by natural means we are forced to resort to dentifrices and tooth brushes to make good the needed natural functions. If there are disease conditions of accidental or temporary causes, change or cure is necessary. We also notice that ordinary functional activity is liable to much traumatic injury as well as incidental wear and is a consequent source of disease. In fact we seem foreordained to be forced to rescue ourselves from actual harm and injury by strenuous efforts to save ourselves from injuries and diseases which continually beset us. There seems to be an inevitable tendency to deterioration as time goes on, and not even the most fortunate can hope to escape these natural hazards and ravages of time and accidental influences. This, however, should not excuse us from making an earnest endeavor to do our best to utilize to the extreme all the science and art we may achieve to prevent waste and injury either materially or physically.

We are learning rapidly to value the natural teeth and the tissues supporting them as we have never before done, we are also learning the reasons why and how the teeth are diseased and lost, and beginning to realize that we must act if we are to save them. This is the day of preventive medicine and dentistry, and it is coming to be a principle in health conservation that all members of the body must be conserved in a condition of health if we are to save the general health. The logic of this ideal demands perfect health for every tooth and its environment in the body. This will require a different standard of health than we have been used to require, as formerly we were satisfied with a part instead of the entire organism.

Since happiness is so dependent on health, what degree of physical health will be sufficient to meet the standards of a complete health certificate; and what is the moral influence involved in this ethical standard? If health is so important to happiness how much should dentistry demand, and how much will the people be willing to do and to give that they may have both health and happiness? In other words, what is the full duty of the dental profession today in a reasonable effort to achieve the maximum for the welfare and happiness of mankind? Who will answer these inquiries and are they reasonable? We should not expect to achieve any such ideals quickly, even though we approve and desire to do so. In the first place we don't know what we should do yet, and then we must learn how to do that which we know we should do; but first of all we shall need to remodel our traditions and throw away a lot of effete and bad habits. All of this will require time and a new line of thinking and work. We can even now visualize the new order and almost realize it at work, but we very well know that it wont work out that way. It will, in all probability, take many more years of patient and disappointing experimentation and countless retracing of steps before we even begin to realize how much we have already built into our fundamentals and really begin to clarify our vision. Does such a dream seem so large as to discourage and leave us hopeless? On the contrary, have we not a great past that should make the joy of living in the present a continual source of inspiration, making all life and effort a continual round of pleasure and happiness because we have hopes and have learned to think with wit and wisdom to guide and inspire us.

